# Etablierung und Umsetzung des Nationalen Aktionsplans Gesundheitskompetenz in Deutschland

**DOI:** 10.1007/s00103-024-04005-1

**Published:** 2025-01-14

**Authors:** Doris Schaeffer, Lennert Griese, Klaus Hurrelmann

**Affiliations:** 1 Fakultät für Gesundheitswissenschaften, Universität Bielefeld, Bielefeld, Deutschland; 2 Hertie School – University of Governance, Berlin, Deutschland

**Keywords:** Gesundheitskompetenz, Gesundheitsinformation, Implementation, Politik, Agenda-Setting, Health literacy, Health information, Implementation, Policy, Agenda setting

## Abstract

Studien zur Gesundheitskompetenz in Deutschland haben bereits vor gut zehn Jahren darauf hingewiesen, dass die Gesundheitskompetenz der Bevölkerung gering ist. Dies war der Anlass für eine Initiative ausgewiesener Expert:innen, einen „Nationalen Aktionsplan Gesundheitskompetenz (NAP-GK)“ für Deutschland nach dem Vorbild anderer Länder zu erarbeiten. In dem vorliegenden Beitrag werden die Entstehung und Erarbeitung des Aktionsplans in Deutschland dargestellt. Es folgt ein Überblick über die dabei durchgeführten Arbeitsschritte und den Inhalt des Aktionsplans. Anschließend wird die Umsetzungsstrategie mit den drei Schritten Diffusion, Dissemination und Implementation dargelegt. In der abschließenden Bilanz werden die Wirkungen des Plans erörtert und die Umsetzungsstrategie kritisch reflektiert. Insgesamt konnten zahlreiche Impulse zum Agenda-Setting und zur Förderung von Gesundheitskompetenz gesetzt werden. Als Herausforderung bleibt eine *nachhaltige* Interventionsentwicklung zur Förderung der Gesundheitskompetenz begleitet durch systematische Forschung.

## Abstract (en)

About ten years ago, studies on health literacy in Germany indicated that population health literacy was low. This prompted a group of distinguished experts to initiate the development of a National Action Plan for Health Literacy (NAP-HL) for Germany, modeled after those of other countries. This article explains the origins and development of the plan in Germany, provides an overview of the steps taken during its creation, and summarizes its content. Subsequently, the transfer strategy, including the three steps of diffusion, dissemination, and implementation, is discussed. The concluding assessment evaluates the plan’s achieved impacts and critically reflects on the implementation strategy. Overall, numerous impulses were provided for agenda setting and the promotion of health literacy. The challenge remains to develop sustainable interventions for strengthening health literacy, accompanied by systematic research.

## Einleitung

Wie die breit gefächerte Literatur und zahlreiche Dokumente der Weltgesundheitsorganisation (WHO) belegen, ist Gesundheitskompetenz – international als „Health Literacy“ bezeichnet – inzwischen zu einem bedeutenden Public-Health-Thema geworden. Definiert wird Health Literacy als „the knowledge, motivation and competences to access, understand, appraise, and apply health information in order to make judgements and take decisions in everyday life concerning healthcare, disease prevention and health promotion“ [1]. Denn zahlreiche Studien zeigen mittlerweile, dass die Gesundheitskompetenz in vielen Ländern – auch in Deutschland – in weiten Teilen der Bevölkerung unzureichend ist [2–6].

Gleichzeitig stellen Gesundheitskompetenz und ein souveräner Umgang mit Gesundheitsinformationen in modernen komplexen Gesellschaften eine immer wichtiger werdende Voraussetzung für eine selbstbestimmte Lebensgestaltung dar [7]. Die voranschreitende digitale Transformation befördert dies in hohem Tempo. Durch sie sind Gesundheitsinformationen zwar heute einfacher zugänglich als noch vor wenigen Jahren, aber zugleich ist es immer schwerer geworden, relevante Informationen in der unüberschaubaren Informationsfülle zu finden und vor allem, sie einschätzen und nutzen zu können [8–12].

In Reaktion auf diese Situation wurden in etlichen Ländern politische Strategien, darunter auch „Nationale Aktionspläne“, zur Förderung der Gesundheitskompetenz aufgelegt [13–15]. Das gilt seit 2018 auch für Deutschland. Über die Entwicklung dieser Strategien und Aktionspläne und auch über die Umsetzung und die ihr zugrundeliegenden Implementationsstrategien und -erfahrungen ist noch immer wenig bekannt. Ziel des vorliegenden Beitrags ist es, die Entstehung und Umsetzung des Nationalen Aktionsplans Gesundheitskompetenz in Deutschland (NAP-GK) darzustellen und zu fragen, welche Erfahrungen damit gesammelt wurden und welche Wirkungen der Plan entfaltet hat. Zuvor wird kurz die Ausgangssituation und die Entwicklung der Gesundheitskompetenzdiskussion in Deutschland betrachtet.^1^

## Gesundheitskompetenz der Bevölkerung in Deutschland – Ausgangssituation

Die Idee, einen NAP-GK zu erarbeiten, entstand im Jahr 2015 mit Bekanntwerden der ersten bevölkerungsbezogenen Studienergebnisse für Deutschland [4, 6, 18]. Bis dahin fand das Thema Gesundheitskompetenz in Deutschland kaum Beachtung.

Anders war die internationale Situation, insbesondere in den USA. Dort existiert bereits seit den 1990er-/2000er-Jahren eine umfangreiche Forschung zur Gesundheitskompetenz, die auf die Diskussion über die gesundheitlichen Folgen geringer Literalität (unzureichende Lese‑, Schreib- und Rechenfähigkeiten) zurückgeht. Denn bereits in den 1990er-Jahren hatten dort durchgeführte Studien wie der „National Adult Literacy Survey“ (NALS) gezeigt, dass geringe Literalität in den USA ein gravierendes gesellschaftliches Problem darstellt [19]. Das ist es bis heute geblieben: Nach wie vor verfügen ca. 20 % der amerikanischen erwachsenen Bevölkerung nicht über grundlegende Lese- und Schreibfähigkeiten. In Deutschland sind es 12 % der Erwachsenen – das entspricht 6,2 Mio. Menschen [20, 21]. Die Studien weisen zudem darauf hin, dass sich geringe Literalität quer durch alle Gesellschaftsschichten zieht, die unteren sozialen Schichten aber besonders trifft, und ebenso, dass sie gesundheitlich folgenreich ist: Denn sie erschwert den Zugang zum Gesundheitssystem und behindert die Compliance, weil Verschreibungen, Therapiehinweise oder Krankheitsinformationen nicht gelesen und verstanden werden können [22, 23].

In der Regel lag diesen wie auch den anschließend entstandenen Studien ein funktionales, auf Literalität begrenztes Verständnis von Health Literacy zugrunde, das in den folgenden Jahren zahlreiche konzeptionelle Weiterentwicklungen erfahren hat. Zu erwähnen sind insbesondere die Überlegungen zum relationalen Charakter von Gesundheitskompetenz, nach denen Gesundheitskompetenz als Ausdruck sowohl persönlicher Fähigkeiten als auch der gegebenen Umgebungs- und Strukturbedingungen zu verstehen ist [24].^2^ Hervorzuheben sind auch die Bemühungen, zu einem weiter gefassten, Public-Health-orientierten Verständnis von Gesundheitskompetenz zu kommen [27].

Durch diese und andere Impulse hat sich Gesundheitskompetenz/Health Literacy inzwischen zu einem umfassenden, multidimensionalen Public Health-orientierten Konzept entwickelt, das nicht nur auf Compliance, sondern auch auf Krankheitsbewältigung/Versorgung, Prävention und Gesundheitsförderung abstellt [1] und dabei auf die Stärkung selbstbestimmter Entscheidungen von Menschen über ihre Gesundheit zielt [7, 28].

Dieses Verständnis prägt auch den ersten Europäischen Health Literacy Survey (HLS-EU) [29], mit dem 2012 faktisch der Startschuss für die Forschung zur Gesundheitskompetenz in Deutschland erfolgte. Der HLS-EU ist aber auch deshalb erwähnenswert, weil mit ihm eine neue, die europäische Diskussion bis heute prägende Definition und ein entsprechendes Erhebungsinstrument^3^ entwickelt wurden und mit ihm – nicht weniger wichtig – erstmals empirische Befunde für Deutschland vorlagen. Allerdings waren sie auf das Bundesland Nordrhein-Westfalen (NRW) beschränkt. Deutschlandweite Daten fehlten somit, und dies motivierte die Entstehung erster Studien zur Gesundheitskompetenz in Deutschland [4, 6, 18].

Dazu gehört auch die erste Studie zur Gesundheitskompetenz der Bevölkerung in Deutschland (Health Literacy Survey Germany 1 – HLS-GER 1 [6]), die 2014 nach dem gleichen methodischen Vorgehen wie der HLS-EU durchgeführt wurde. Die Ergebnisse dieser Studie sorgten sowohl in der gesundheitswissenschaftlichen als auch der gesundheitspolitischen Diskussion für eine nachhaltige Veränderung der Aufmerksamkeit für das Thema. Denn sie zeigten, dass mehr als die Hälfte der Bevölkerung in Deutschland – konkret 54,3 % – eine geringe Gesundheitskompetenz aufweist. Sie verdeutlichten zudem, dass Gesundheitskompetenz ungleich verteilt ist und geringe Gesundheitskompetenz u. a. mit niedriger Bildung, niedrigem Sozialstatus und einem höheren Lebensalter assoziiert ist [6]. Anders formuliert: Menschen mit diesen Merkmalen gehören zu den sogenannten „vulnerablen“ Gruppen, die besonders große Schwierigkeiten im Umgang mit Gesundheitsinformationen haben und bei der Förderung von Gesundheitskompetenz spezielle Beachtung erhalten sollten. Das ist umso wichtiger, als geringe Gesundheitskompetenz mit zahlreichen negativen Konsequenzen einhergeht, die von ungesünderen Verhaltensweisen über verminderte Nutzung von Präventionsangeboten, ein höheres Krankheitsrisiko bis hin zu einer intensiveren Nutzung des Gesundheitssystems reichen und erhebliche Kosten verursachen [3, 31–33].

Ähnliche Tendenzen zeigen sich auch in anderen Ländern (z. B. [2]) sowie in nachfolgenden Studien zur Gesundheitskompetenz der Bevölkerung und einzelner Bevölkerungsgruppen in Deutschland (z. B. [34–37]).

## Entstehung und Aufbau des Nationalen Aktionsplans in Deutschland

Die dargestellten Erkenntnisse zur Gesundheitskompetenz haben international die Entstehung zahlreicher Strategien und Nationaler Aktionspläne zur Verbesserung der Gesundheitskompetenz nach sich gezogen. Auch in Deutschland bildete sich unter dem Eindruck der ersten Studienergebnisse 2015 eine Initiative zur Erarbeitung eines Nationalen Aktionsplans Gesundheitskompetenz. Anders als üblich wurde sie nicht durch eine politische Instanz initiiert, sondern entstand unter der Federführung von Wissenschaftler:innen der Universität Bielefeld als zivilgesellschaftliche Initiative und setzte sich aus einer Gruppe von ausgewiesenen nationalen und internationalen Expert:innen aus unterschiedlichen wissenschaftlichen Disziplinen, Institutionen der Gesundheitsversorgung und der Gesundheitspolitik zusammen.

Ende 2015 startete die Erarbeitung des NAP-GK. Sie beruhte auf mehreren Arbeitsschritten [16] und begann mit einer Recherche und Analyse vorliegender Aktionspläne sowie einem Review der vorliegenden Literatur und der existenten Studienbefunde. Es folgten ausgiebige Diskussionen der daraus erwachsenen Konsequenzen für die Kontur und den Inhalt des zu erarbeitenden Aktionsplans für Deutschland – dies im Plenum sowie in unterschiedlichen Arbeitsgruppen der NAP-GK-Expertengruppe. Sie mündeten in der Erstellung eines ersten Entwurfs für den NAP-GK. Daran schloss sich ein umfangreicher Diskussions- und Konsentierungsprozess an: In einem ersten Schritt wurde ein Workshop mit Stakeholdern und Vertreter:innen der Verbände im Gesundheits- und Bildungswesen sowie den Mitgliedern der „Allianz für Gesundheitskompetenz“ (Initiative des Bundesministeriums für Gesundheit, BMG) durchgeführt, um die Empfehlungen des Plans in den unterschiedlichen Handlungsfeldern zu diskutieren und zu ergänzen. Ein zweiter Workshop richtete sich gezielt an Selbsthilfe- und Patientenorganisationen mit dem Ziel, den Entwurf des NAP-GK aus ihrer Perspektive zu kommentieren. Zusätzlich wurden Einzelgespräche mit weiteren relevanten Interessen- und Arbeitsgruppen sowie einzelnen Stakeholdern durchgeführt. Alle Schritte wurden protokolliert, diskutiert und wichtige relevante Ergebnisse anschließend eingearbeitet. Nach mehrfacher Überarbeitung der Rohfassung erfolgte im Spätherbst 2017 die Finalisierung des NAP-GK.

Der innerhalb von knapp zwei Jahren erarbeitete NAP-GK enthält 15 aufeinander abgestimmte Empfehlungen in vier Handlungsbereichen, wie die Gesundheitskompetenz in Deutschland gefördert werden kann und welche Maßnahmen dazu angestoßen werden sollten. Die Empfehlungen zielen auf unterschiedliche Bereiche des gesellschaftlichen Lebens und widmen sich zu gleichen Teilen der Stärkung der persönlichen Gesundheitskompetenz wie der Verbesserung der Umgebungs- und Strukturbedingungen.

Im Mittelpunkt des *ersten Handlungsbereichs* stehen die alltäglichen Lebenswelten. Die Empfehlungen konzentrieren sich auf die Stärkung der Gesundheitskompetenz im Erziehungs- und Bildungsbereich, der Wohnumgebung, der Arbeitswelt, der Kommune, in den Medien und im Freizeit- und Konsumbereich.

Der zweite *Handlungsbereich* widmet sich dem Gesundheitssystem. Aufgrund seiner Komplexität, Instanzenvielfalt und Intransparenz stellt es sehr hohe Anforderungen an die Nutzenden. Empfohlen wird, es zu einem gesundheitskompetenten, nutzerfreundlichen und patientenzentrierten System weiterzuentwickeln und dazu die Navigation, Kommunikation, Information und Partizipation zu verbessern.

Der *dritte Handlungsbereich* befasst sich mit dem Leben mit chronischen Erkrankungen, die sich immer weiter ausbreiten. Sie gehen mit zahlreichen Bewältigungsherausforderungen an die Erkrankten und ihre Familien einher. Die Empfehlungen zielen darauf ab, den Betroffenen einen gesundheitskompetenten Umgang mit der Krankheit und ihren zahlreichen Begleiterscheinungen zu ermöglichen, ihre Fähigkeit zum kompetenten Selbstmanagement zu stärken und das Alltagsleben mit chronischer Erkrankung zu erleichtern.

Der *vierte Handlungsbereich* konzentriert sich auf die Verbesserung (und den Ausbau) der *Forschung *zur Gesundheitskompetenz [38].

Der NAP-GK wurde im Februar 2018 dem damaligen Gesundheitsminister, der zugleich Schirmherr des Vorhabens war, übergeben. Der Plan richtet sich vor allem an die Politik und an relevante Akteur:innen im Gesundheitssystem und in anderen Bereichen der Gesellschaft (Erziehungs- und Bildungssystem, Freizeitbereich, Arbeitswelt etc.). Sein übergeordnetes Ziel besteht darin, gemäß der Prämisse „Health Literacy in all Policies“ ein Kooperationsbündnis aller Bereiche der Gesellschaft zu initiieren und so ein umfassendes Vorgehen bei der Förderung der Gesundheitskompetenz zu ermöglichen.

## Umsetzung des Nationalen Aktionsplans in Deutschland

Mit der Veröffentlichung des NAP-GK 2018 trat die Arbeit der Expertengruppe in eine neue Phase ein und begann die Umsetzung des NAP-GK. Mit ihr wurde weitgehend Neuland betreten. Denn zur damaligen Zeit lagen erst wenige Erfahrungen vor, wie die Umsetzung solcher Aktionspläne in die komplexen Strukturen der Politik und der Praxis erfolgen kann. Auch Diskussionen über Implementationsstrategien und -erfahrungen existierten nicht. Sie entstehen erst langsam (z. B. [39]). Vielfach waren die bis dato existenten Aktionspläne zudem in Ländern mit einem stärker staatlich geprägten Gesundheitssystem entstanden, in denen die Umsetzung von Empfehlungen prinzipiell dadurch möglich ist, dass von übergeordneten Instanzen Richtlinien formuliert werden, deren Umsetzung überprüft und eingefordert werden kann. Mittlerweile wird bezweifelt, dass solche Top-Down-Strategien allein erfolgversprechend sind ([40], auch [31]). Analysen der Umsetzung politischer Programme oder gesetzlicher Regelungen zufolge ist anzunehmen, dass sie selbst in zentralistisch gesteuerten Systemen nur sehr bedingt wirkungsvoll sind [40]. Weil Deutschland kein zentral gesteuertes Gesundheitssystem besitzt, sondern als sogenannter „konservativer“ Wohlfahrtsstaat fast alle seine Sicherungs- und Versorgungssysteme nach dem Subsidiaritätsprinzip gestaltet hat, kam für die Umsetzung ohnehin keine Top-Down-Strategie in Frage. Im deutschen Gesundheitssystem kann der Staat nur politische Grundsätze formulieren, deren Konkretisierung und Umsetzung durch eigenständige, selbstorganisierte, relativ autonome Organisationen und Verbände erfolgt.

Für die Umsetzung des NAP-GK erwuchs daraus die Notwendigkeit, nicht nur politische Entscheidungsträger, sondern auch die entsprechenden Instanzen, Organisationen, Verbände und Akteur:innen der Selbstverwaltung im Gesundheitssystem und in anderen Infrastrukturbereichen in die Umsetzung und Realisierung der Empfehlungen einzubeziehen. Damit bot sich eine eher Bottom-up ausgerichtete, auf Kooperation setzende Umsetzungsstrategie an, um Institutionen aus möglichst vielen Bereichen zur Umsetzung des Aktionsplans zu motivieren. Das sprach für eine auf Kooperation setzende Implementationsstrategie mit „upstream and downstream actors“ aus unterschiedlichen Bereichen [41].

Unter Rückgriff auf implementationswissenschaftliche Überlegungen wurde die Umsetzung des NAP-GK zudem nicht als ein einmaliger Vorgang, sondern als *fortlaufender Prozess* konzipiert [42]. Dafür spricht, dass die Umsetzung von Aktionsplänen oder politischen Strategien fast immer auch mit der Einführung von Innovationen verbunden ist, deren Übernahme sich selten ad hoc, sondern meist schrittweise vollzieht. Sie stößt zudem oft zunächst auf Widerstand [41], dessen Ausräumung Zeit erfordert.

**Abb. 1:**
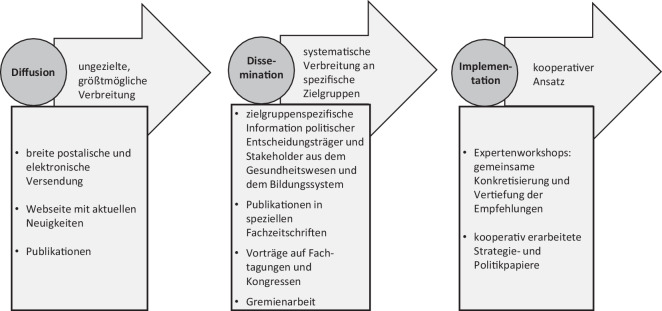
Implementationsstrategie des Nationalen Aktionsplans Gesundheitskompetenz (NAP-GK). Erweiterte Darstellung aus [17], © 2020 von den Autoren. Lizenznehmer MDPI, Basel, Schweiz. Dieser Artikel ist ein Open-Access-Artikel, der unter den Bedingungen der Creative Commons Attribution (CC BY) Lizenz verbreitet wird (http://creativecommons.org/licenses/by/4.0/)

Als weiteres wichtiges Merkmal der Umsetzungsstrategie des NAP-GK ist anzuführen, dass sie als Kontinuum von drei unterschiedlichen, aufeinander aufbauenden und sich überlappenden Schritten von Diffusion, Dissemination und Implementation angelegt wurde ([43, 44]; Abb. 1).

### Erster Schritt der Umsetzung: Diffusion

Der erste Schritt „Diffusion“ zielte darauf, eine möglichst breite Streuung des NAP-GK und ein an der Umsetzung interessiertes Klima zu schaffen. Dazu wurde der Aktionsplan auf unterschiedlichen Kanälen publik gemacht. Ausgangspunkt war eine Großveranstaltung, an der Akteur:innen aus Politik, Selbstverwaltung, Medien und Wissenschaft teilnahmen. Anschließend wurde der Aktionsplan umfangreich postalisch und digital distribuiert und auf Tagungen vorgestellt. Besonders die Website^4^ hat sich als wichtiges Medium zur Distribution des NAP-GK erwiesen. Sie liefert Hintergrundinformationen über den NAP-GK, das Leitungsteam, die Expertengruppe und die Koordinierungsstelle sowie geplante und durchgeführte Veranstaltungen und sonstige Aktivitäten. Auf diese Weise gelang es, den NAP-GK in unterschiedlichen Netzwerken auch in vielen Verbänden und Vereinen bekannt zu machen. Zugleich wurden zahlreiche Presseberichte und Mediennachrichten über den Aktionsplan gestreut.

### Zweiter Schritt der Umsetzung: Dissemination

Bei der parallel eingeleiteten Dissemination ging es um eine gezielte Verbreitung des NAP-GK an ausgewählte, wichtige Zielgruppen und Stakeholder, so u. a. Politiker:innen auf Bundes- und Landesebene, Leitungskräfte von Verbänden und Organisationen des Gesundheitswesens sowie von Wohlfahrtsorganisationen oder Einrichtungen des Erziehungs- und Bildungssektors und Stiftungen. Ziel war es, sie über den NAP-GK und seine Empfehlungen zu informieren, bei ihnen Adoptionsbereitschaft zu wecken und sie zu motivieren, sich in ihrem Handlungsbereich für die Förderung von Gesundheitskompetenz zu engagieren. Dazu erfolgten Publikationen in entsprechenden Fachzeitschriften, ebenso zahlreiche Vorträge über den NAP-GK auf Tagungen, unterschiedlichsten Fachveranstaltungen und Kongressen im Gesundheitssystem und in anderen relevanten Gesellschaftsbereichen. Zudem wurden zahlreiche Beiträge in Fachzeitschriften zum NAP-GK erstellt und veröffentlicht.

### Dritter Schritt der Umsetzung: Implementation

Ein dritter Schritt zielte auf die Implementation des NAP-GK in für die Förderung von Gesundheitskompetenz als wichtig erachtete Handlungsbereiche. Im Mittelpunkt dieses Schritts standen a) kooperative Workshops mit Stakeholdern und Vertreter:innen aus der Politik, Wissenschaft und Praxis und b) eine Zusammenarbeit mit relevanten Netzwerken nach dem Motto „Health Literacy in all Policies“.

#### a) Workshops

**Abb. 2:**
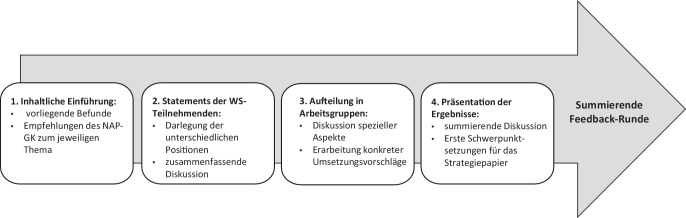
Ablaufmuster der Workshops zum Nationalen Aktionsplan Gesundheitskompetenz (NAP-GK). Eigene Abbildung

Die Workshops bilden das Kernelement der Umsetzungsstrategie des NAP-GK. Ziel der Workshops sollte es sein, eine kooperative Weiterbearbeitung der Empfehlungen des NAP-GK in konkrete, direkt umsetzbare Handlungsschritte zu initiieren. Intention war es, auf diese Weise eine Identifikation mit dem NAP-GK und dessen Adoption zu erreichen und die beteiligten Akteur:innen zu motivieren, sich in ihrem Wirkungsfeld für die Umsetzung zu engagieren. Als Ergebnis der Workshops wurden jeweils *Strategiepapiere *erarbeitet und anschließend distribuiert.^5^ Insgesamt wurden neun von der Koordinierungsstelle des NAP-GK organisierte Workshops mit unterschiedlichen Akteur:innen durchgeführt, an denen jeweils etwa 25–30 Teilnehmende mitwirkten. Die Workshops folgten dabei einem festen Ablaufmuster (Abb. 2).

Von den Workshops wurden ausführliche Protokolle erstellt, die als Datenmaterial für die anschließende kooperative Erarbeitung eines Strategiepapiers dienten. Dazu wurden die wichtigsten Ergebnisse der Workshops anschließend zusammengefasst und erste hypothesenartige Kernaussagen festgelegt. Auf dieser Basis wurde ein von der Leitung der Workshops verantwortetes Strategiepapier entworfen. Es wurde den Workshopteilnehmenden zur Kommentierung zugesendet und anschließend meist mehrfach überarbeitet, bis alle einer Publikation zustimmten.

Die Strategiepapiere wurden den Teilnehmenden für ihre Netzwerke zur Verfügung gestellt und parallel über die Website des NAP-GK distribuiert. Teilweise wurden sie ergänzend in Zeitschriften veröffentlicht. Die Strategiepapiere stießen in der Regel auf große Resonanz. In einigen Organisationen führten sie zu intensiven Diskussionen. Sie stimulierten außerdem die Identifikation mit dem NAP-GK und auch die Umsetzungsbereitschaft, was sich u. a. in den Anfragen zu Vorträgen und eigenen Projektplanungen zeigte. Bis heute, nach rund siebenjährigem Bestehen des NAP-GK, ist eine breite Palette an Praxisinitiativen zu finden, die auf den Aktionsplan Bezug nehmen.

#### b) Kooperation mit Netzwerken

Ein weiterer Bestandteil der kooperativen Implementationsstrategie bestand in der Zusammenarbeit mit Netzwerken und Mitwirkung in Gremien oder Arbeitsgruppen. Dazu gehörten z. B. die Arbeitsgruppe Gesundheitskompetenz im Nationalen Krebsplan, eine gleichnamige Gruppe in einem Pflegeverband, Beiräte von Institutionen, die entstehenden Netzwerke zur Gesundheitskompetenz wie etwa die AG Gesundheitskompetenz im Deutschen Netzwerk Versorgungsforschung und in der Deutschen Gesellschaft für Sozialmedizin und Prävention oder das Deutsche Netzwerk Gesundheitskompetenz (DNGK). Eine besondere Bedeutung hat die Kooperation mit der „Allianz Gesundheitskompetenz“, die 2017 vom BMG gegründet wurde und das Ziel hat, im Gesundheitswesen Praxisprojekte zur Förderung der Gesundheitskompetenz anzustoßen. Mit dieser Programmatik war und ist die Allianz Gesundheitskompetenz für die Umsetzung des Aktionsplans besonders wichtig. Im Gegenzug konnten die Mitglieder der Allianz dabei unterstützt werden, Ideen für Praxisprojekte zu entwickeln und zu realisieren, denn dazu fehlte es zu Beginn vielfach an Fachexpertise.

## Einschnitt durch die Corona-Krise

Nach gut drei Jahren war der NAP-GK zu einer Art Referenzwerk für die Förderung von Gesundheitskompetenz in Deutschland geworden und hatte zur Entstehung zahlreicher Initiativen beigetragen – in der Wissenschaft, wie der Praxis oder der Politik. Dann kam die COVID-19-Pandemie, die in fast allen Bereichen gesellschaftlichen Lebens zu einer abrupten Zäsur geführt hat.

Auch die Implementationsstrategie des NAP-GK geriet durch sie an Grenzen und musste modifiziert werden, zumal auch die bis dahin erfolgreiche Karriere des Themas Gesundheitskompetenz unerwartet ins Trudeln geriet. Erstaunlich war dies deshalb, weil die Pandemie rasch deutlich zeigte, wie wichtig es in gesundheitlichen Krisen ist, im richtigen Moment die richtigen Informationen zum Umgang mit der unvertrauten Situation zu finden, zu verstehen, einzuordnen und für das eigene Gesundheitsverhalten nutzen zu können. Gerade während der Pandemie wurde die Stärkung der Gesundheitskompetenz daher wichtiger denn je, zumal die Bevölkerung ad hoc mit umfangreichen Informationsherausforderungen konfrontiert war, ohne auf gesichertes Wissen zurückgreifen und ohne sich auf den üblichen Informationspfaden bewegen zu können.

Zugleich zeigten die Ergebnisse der zweiten Studie zur Gesundheitskompetenz der Bevölkerung in Deutschland (HLS-GER 2) aus den Jahren 2019/2020, dass sich die Gesundheitskompetenz in Deutschland seit der ersten Studie verschlechtert hat. Auch die ungleiche Verteilung von Gesundheitskompetenz hatte sich verstärkt [45]. Es war also davon auszugehen, dass es um die Gesundheitskompetenz in Deutschland zum Zeitpunkt der Pandemie nicht gut bestellt war. Erste Studien zur coronaspezifischen Gesundheitskompetenz kamen alsbald zu ähnlichen Ergebnissen [46].

Für die NAP-GK-Expertengruppe bedeutete dies, dass die Förderung von Gesundheitskompetenz und die Umsetzung des NAP-GK intensiviert werden mussten. Doch hatten sich die Umsetzungsbedingungen durch die Corona-Pandemie und ihre Begleiterscheinungen wie Kontaktbeschränkungen etc. völlig verändert. Vor allem die Workshopstrategie und die Kooperation mit Gremien und Arbeitsgruppen war kaum noch realisierbar, weil keine persönlichen Zusammenkünfte mehr möglich waren. Daher wurde die Umsetzungsstrategie geändert: Statt Workshops wurde vor allem auf Positionspapiere und Policy Briefs gesetzt, die kooperativ in unterschiedlichen Teams digital erarbeitet wurden. Es entstanden insgesamt fünf solcher Dokumente.

Parallel bildete sich in dieser Zeit eine internationale Arbeitsgruppe zu „Health Literacy Policies“ und deren Implementation im „WHO Action Network on Measuring Population and Organizational Health Literacy“ (M-POHL), an der Vertreter:innen des NAP-GK mitwirkten und zur Entstehung eines entsprechenden Policy Guides beitrugen [39].

Zum Ende der Pandemie hin wurde deutlich, dass eine abermalige Modifikation der Umsetzungsstrategie des NAP-GK erforderlich ist. Sie wird aktuell diskutiert und zusammen mit dem Deutschen Netzwerk für Gesundheitskompetenz (DNGK) erstellt.

## Kritische Bilanz bisher erreichter Ziele – Lessons Learned

Insgesamt ist es in den sieben Jahren des Bestehens des NAP-GK gelungen, viele der Empfehlungen in die öffentliche Diskussion zu tragen und zahlreiche Impulse zum Agenda-Setting und zur Initiierung von Projekten zur Förderung von Gesundheitskompetenz zu setzen. Die wichtigsten mit dem NAP-GK und seiner Entstehungs- und Umsetzungsstrategie gesammelten Erfahrungen sollen abschließend dargestellt und summierend reflektiert werden.

### 1. Zivilgesellschaftliche Initiative.

Zu den Besonderheiten des deutschen NAP-GK gehört, dass er nicht durch eine von der Regierung eingesetzte Kommission erarbeitet wurde, sondern von einer Gruppe ausgewiesener und von der Wichtigkeit des Themas überzeugter Akteur:innen. Er kann somit als Beispiel dafür gelten, dass zivilgesellschaftliche Initiativen entscheidend zur Entwicklung neuer Politikfelder und zum Kapazitätsaufbau eines neuen gesundheitlich relevanten Themas beitragen können. Zugleich hat diese Konstruktion Vor- und Nachteile. Einerseits war auf diese Weise die Unabhängigkeit und Neutralität gewährleistet, ebenso hohe Motivation. Andererseits birgt diese Konstruktion das Risiko politischer Distanz und schwacher politischer Resonanz und Unterstützung in sich. Sie konnte durch die frühe Übernahme der Schirmherrschaft des Bundesgesundheitsministers über den NAP-GK umgangen werden. Diese verlieh dem NAP-GK besonders in der Anfangszeit eine gewisse politische Legitimität und ein entsprechendes Ansehen. Mit den sich in der Folgezeit vollziehenden Ministerwechseln veränderte sich dies. Doch war der NAP-GK inzwischen zu einer festen Größe geworden, was sich u. a. daran zeigt, dass er in einem kürzlich erschienenen Bericht der Organisation für wirtschaftliche Zusammenarbeit und Entwicklung (OECD), in dem anhand ausgewählter Länderbeispiele unterschiedliche Initiativen zur Verbesserung der Gesundheitskompetenz dargestellt werden, explizit aufgegriffen wurde [47].

### 2. Kooperativer Ansatz und Klärung von Missverständnissen.

Der kooperative Ansatz bei der Entwicklung und Umsetzung hat wesentlich zum Erfolg des NAP-GK beigetragen: Er ermöglichte die Einbindung vieler wichtiger Akteur:innen, Stakeholder und Interessengruppen. Dadurch gelang es, das Thema in der Fachdiskussion zu platzieren und ihm Akzeptanz zu verschaffen. Zugleich trug dieser Ansatz zur Ausräumung anfänglich bestehender Vorbehalte gegenüber dem Konzept der Gesundheitskompetenz bei. Denn vor allem die Übersetzung von „Health Literacy“ mit „Gesundheitskompetenz“ stieß bei den Beteiligten zunächst auf Missverständnisse, weil sie mit der seit Jahren intensiv geführten Debatte über Gesundheitsförderung und auch über den Kompetenzbegriff und die Kompetenzmessung kollidierte und umfangreiche Klärungsbemühungen erforderte. So wurde beispielsweise der Begriff Gesundheitskompetenz zu Beginn vielfach mit Gesundheitsförderung und der Fähigkeit, sich gesundheitsbewusst zu verhalten, gleichgesetzt. Der für Gesundheitskompetenz zentrale Aspekt – die Fähigkeit zum Umgang mit gesundheitsrelevanten Informationen – wurde vielfach übersehen. Solche Passungsprobleme mit etablierten Konzepten und daraus erwachsener Klärungsbedarf sind keine Seltenheit bei der Einführung von neuen Konzepten und stellen keine nationale Besonderheit dar [48]. Auch international stieß das Konzept zunächst auf Irritation und teilweise auch Aversion [13]. Ähnliches gilt für die den populationsorientierten Studien zugrundeliegende Erhebungsmethodik. Auch sie wurde zunächst (und wird) kritisch betrachtet. Beobachtet werden konnte, dass sich viele solcher Passungsprobleme im Lauf der Zeit abschliffen und auch die Widerstände gegen eine Adoption des Konzepts und der Erhebungsmethodik nachließen. Dennoch bedürfen sie nach wie vor der Beachtung, denn die bestehenden Divergenzen und Nahtstellen zu angrenzenden Konzepten oder die Auseinandersetzung über unterschiedliche Erhebungsmethoden sind bisher keineswegs beendet (z. B. [15, 49]).

### 3. Umsetzung frühzeitig mitdenken.

Insgesamt zeigen die Erfahrungen mit dem NAP-GK, dass es nicht reicht, Aktionspläne und/oder andere Strategien zur Förderung von Gesundheitskompetenz zu erarbeiten, sondern wichtig ist, den sich anschließenden Schritt – die Umsetzung – frühzeitig mitzudenken und zu planen. Dies geschieht weitaus zu wenig. Egal, ob bei Aktionsplänen, innovativen Politikstrategien, Gesetzen oder Empfehlungen aus Expertenkommissionen – oft wird auf naturwüchsige Umsetzung gesetzt. Doch ist diese Strategie selten erfolgreich und führt zu zahlreichen Umsetzungsdefiziten, wie auch die Literatur aufzeigt (ex. [40, 44]). Die mit dem NAP-GK gesammelten Erfahrungen bestätigen dies einmal mehr und verdeutlichen, dass vorliegende implementationswissenschaftliche Erkenntnisse offenbar noch größere Beachtung erfahren müssen.

Betrachtet man die der NAP-GK Umsetzung zugrundliegenden Schritte, zeigen die gesammelten Erfahrungen, wie essenziell die Schritte Diffusion und Dissemination sind, um neue Themen und Programme bekannt zu machen und um Umsetzungsinteresse und -motivation zu erzeugen. Zugleich wurde retrospektiv deutlich, dass beide Schritte, obschon sehr viel Energie auf sie verwendet wurde, intensivierungsbedürftig sind. Wie Studien mittlerweile zeigen, ist die Bekanntheit des Gesundheitskompetenz-Konzepts, z. B. bei den Gesundheitsprofessionen und damit bei wichtigen Instanzen der Förderung von Gesundheitskompetenz, nach wie vor begrenzt [50], sodass es weiterer und breitenwirksamerer Aufklärung bedarf.

### 4. Kooperative Workshops.

Der dritte Schritt, die Implementation durch kooperative Workshops zur Operationalisierung der Empfehlungen des NAP-GK mit Politiker:innen und Stakeholdern bildete das Kernstück der Umsetzung. Auch er hat sich als erfolgreich erwiesen, wie allein die Resonanz in den Feedback-Runden und auch die unerwartete Identifikation mit den Strategiepapieren gezeigt haben. Die Durchführung der Workshops und insbesondere die Rekrutierung der Teilnehmenden entpuppten sich zuweilen allerdings als sehr aufwändig und anspruchsvoll – aus terminlichen und organisatorischen Gründen. Ähnlich war es auch während der Corona-Pandemie bei den digitalen Meetings zur Erarbeitung der Policy Briefs bzw. Positionspapiere. Hinzu kam, dass Repräsentanten der Politik nur schwer für eine Teilnahme zu gewinnen waren. Stakeholder machten daher die Mehrheit der Teilnehmenden aus. Zudem zeigte sich bald, dass einmalige Workshops nicht ausreichen und lediglich die Funktion eines „Appetizers“ einnehmen. Sie können die Innovations- und Implementationsbereitschaft zwar anregen, aber keine zeitstabilen Effekte erzeugen. Wichtig ist deshalb – wie die Erfahrungen bestätigten – ein langfristig angelegtes, prozessuales Vorgehen mit Wiederholungsschlaufen und Vertiefungen in unterschiedlichen Formaten, zumal auch die Stimulierung und Umsetzung von Innovationen im Wirkungsfeld der Teilnehmenden selten reibungslos verläuft und Zeit benötigt [42, 44]. Zugleich sollte der kooperative Ansatz bei allen Bemühungen unbedingt beibehalten werden. Er ist zwar aufwändig, hat sich jedoch bewährt und als wirkungsvoll erwiesen. Das gilt auch für die Kooperation mit Gremien, Arbeitsgruppen und Netzwerken.

### 5. Stärker einzubeziehende Bereiche.

Die Dissemination und Implementation des NAP-GK hat sich vornehmlich auf das Gesundheitssystem konzentriert. Das Erziehungs- und Bildungssystem und weitere wichtige Bereiche (Kommune, Ernährung) wurden tendenziell zu wenig einbezogen. Ursache dafür ist, dass dies Ressourcen verlangt, die dem NAP-GK-Projekt nicht zur Verfügung standen. Die Themen „vulnerable Gruppen“ und damit einhergehend „Vermeidung von Ungleichheit“ haben zwar eine bedeutende Rolle bei der Implementation gespielt – allein vier von den neun Workshops widmeten sich einzelnen vulnerablen Gruppen und Ungleichheit wurde wiederum zum wichtigen Querschnittthema erhoben – dennoch sollten auch sie künftig noch intensiver aufgegriffen werden.

### 6. Monitoring ermöglichen.

Wünschenswert wäre zudem eine formative Evaluation zur Ermittlung der Wirkungen des NAP-GK gewesen. Auch sie setzt allerdings mehr Ressourcen und Kapazitäten voraus. Gleichzeitig können vorliegende Bevölkerungsbefragungen zur Evaluation genutzt werden, insbesondere wenn sie als regelmäßiges Monitoring angelegt sind – wie auch von der WHO betont wird [51]. Ein solches Monitoring zu ermöglichen, ist nach wie vor eine der Zukunft vorbehaltene Aufgabe. Denn bislang liegen in Deutschland kaum Wiederholungsbefragungen zur Gesundheitskompetenz vor, die vergleichende Trendanalysen erlauben und zur Evaluation herangezogen werden können. Die wenigen vorliegenden vergleichbaren populationsorientierten Daten [37, 45, 46] deuten jedoch bereits an, dass ein solches Monitoring wichtig sein dürfte, um Veränderungen der Gesundheitskompetenz im Zeitverlauf beobachten und Fördermaßnahmen bewerten zu können.

## Fazit

Angesichts anhaltender alter und neuer Krisen mit ihren unterschiedlichen gesellschaftlichen und gesundheitlichen Auswirkungen und Ungewissheiten steigt die Bedeutung von Gesundheitskompetenz weiter. Sah es zunächst so aus, als würde Gesundheitskompetenz rasch zu einem neuen Politikfeld werden, sind inzwischen Zweifel angebracht. Zwar stößt das Thema Gesundheitskompetenz in der Wissenschaft und auch in der Praxis mittlerweile auf bemerkenswerte Aufmerksamkeit. Auf politischer Ebene entspricht die Resonanz jedoch nicht der gesellschaftlichen Bedeutung des Themas, obschon geringe Gesundheitskompetenz in Deutschland nach wie vor ein nicht zu unterschätzendes Public-Health-Problem darstellt, das große Teile der Bevölkerung betrifft. Daher bleibt es eine zentrale Aufgabe, sich weiterhin für ein nachhaltiges Agenda-Setting und eine systematische Interventions- und Forschungsentwicklung zur Gesundheitskompetenz mit ausreichenden finanziellen Ressourcen zu engagieren.

Um dies anzustoßen und die weitere Arbeit mit dem Nationalen Aktionsplan auf eine noch breitere Basis zu stellen, ist die zentrale Koordination ab 2025 von der Universität Bielefeld und der Hertie School, Berlin, an das Deutsche Netzwerk für Gesundheitskompetenz übergegangen.

## Footnotes

1 Der vorliegende Beitrag nimmt Bezug auf zwei Publikationen zur Entwicklung und Umsetzung des NAP-GK [16, 17].

2 Umgebungs- und Strukturbedingungen bilden den Ausgangspunkt für das vergleichsweise junge Konzept der „Organisationalen Gesundheitskompetenz“, das auf die Bedeutung der Gesundheitseinrichtungen (und -professionen) für die Informationsvermittlung und Förderung von Gesundheitskompetenz abhebt (z. B. [25, 26]).

3 Das im HLS-EU entwickelte Erhebungsinstrument HLS-EU‑Q misst die Gesundheitskompetenz über die Abfrage selbsteingeschätzter Schwierigkeiten bei der Bewältigung von Aufgaben beim Finden, Verstehen, Beurteilen und Anwenden von Gesundheitsinformationen in den Bereichen Krankheitsbewältigung/Versorgung, Prävention und Gesundheitsförderung (zum Thema Selbsteinschätzungs- versus performanzbasierte Erhebungsinstrumente siehe z. B. Stock et al. [30]).

4 www.nap-gesundheitskompetenz.de.

5 Eine Übersicht über alle Strategiepapiere des NAP-GK findet sich unter https://www.nap-gesundheitskompetenz.de/aktionsplan/strategiepapiere/; Zugriff: 10.12.2024.

## References

[1] Sørensen K, van den Broucke S, Fullam J et al (2012) Health literacy and public health. A systematic review and integration of definitions and models. Bmc Public Health 12(1):80. 10.1186/1471-2458-12-80 22276600 10.1186/1471-2458-12-80 PMC3292515

[2] Baccolini V, Rosso A, Di Paolo C et al (2021) What is the Prevalence of Low Health Literacy in European Union Member States? A Systematic Review and Meta-analysis. J Gen Intern Med 36:753–761. 10.1007/s11606-020-06407-8 33403622 10.1007/s11606-020-06407-8 PMC7947142

[3] The HLS_19_ Consortium of the WHO Action Network M-POHL (Hrsg) (2021) International report on the methodology, results, and recommendations of the European Health Literacy Popula-tion Survey 2019-2021 (HLS19) of M-POHL. Austrian National Public Health Institute, Vienna

[4] Jordan S, Hoebel J (2015) Gesundheitskompetenz von Erwachsenen in Deutschland. Bundesgesundheitsbl 58:942–950. 10.1007/s00103-015-2200-z 10.1007/s00103-015-2200-z 26227894

[5] Nakayama K, Osaka W, Togari T et al (2015) Comprehensive health literacy in Japan is lower than in Europe: a validated Japanese-language assessment of health literacy. Bmc Public Health 15:505. 10.1186/s12889-015-1835-x 26001385 10.1186/s12889-015-1835-x PMC4491868

[6] Schaeffer D, Berens E‑M, Vogt D (2017) Health Literacy in the German Population. Dtsch Ärztebl Int 114:53–60. 10.3238/arztebl.2017.0053 28211318 10.3238/arztebl.2017.0053 PMC5319379

[7] Kickbusch I, Maag D, Saan H (2005) Enabling healthy choices in modern health societies. Background paper for the European Health Forum. Badgastein, Bd. 2005

[8] Afful-Dadzie E, Afful-Dadzie A, Egala SB (2023) Social media in health communication: A literature review of information quality. Health Inf Manag 52:3–17. 10.1177/1833358321992683 33818176 10.1177/1833358321992683

[9] Baumann E, Czerwinski F, Rosset M, Seelig M, Suhr R (2020) Wie informieren sich die Menschen in Deutschland zum Thema Gesundheit? Erkenntnisse aus der ersten Welle von HINTS Germany. Bundesgesundheitsblatt 63:1151–1160. 10.1007/s00103-020-03192-x 10.1007/s00103-020-03192-x 32666180

[10] Eysenbach G (2002) Infodemiology: the epidemiology of (mis)information. Am J Med 9:763–765 10.1016/s0002-9343(02)01473-0 12517369

[11] Khaleel I, Wimmer BC, Peterson GM et al (2020) Health information overload among health consumers: A scoping review. Patient Educ Couns 103:15–32. 10.1016/j.pec.2019.08.008 31451363 10.1016/j.pec.2019.08.008

[12] Okan O, Messer M, Levin-Zamir D et al (2023) Health literacy action framework for health emergencies and infodemics. ISU 43:115–130. 10.3233/ISU-230193

[13] Weishaar H, Hurrelmann K, Okan O, Horn A, Schaeffer D (2019) Framing health literacy: A comparative analysis of national action plans. Health Policy 123:11–20. 10.1016/j.healthpol.2018.11.012 30527962 10.1016/j.healthpol.2018.11.012

[14] van den Broucke S (2019) Capacity building for health literacy. In: Okan O, Bauer U, Levin-Zamir D, Pinheiro P, Sørensen K (Hrsg) International Handbook of Health Literacy. Policy Press, S 705–720

[15] Rowlands G, Trezona A, Russell S et al (2019) What is the evidence on the methods frameworks and indicators Used to Evaluate Health Literacy Policies Programmes and Interventions at the regional national and organizational levels? Health Evidence Network synthesis report Bd. 65. World Health Organization, Geneva 31693320

[16] Schaeffer D, Gille S, Vogt D, Hurrelmann K (2021) National Action Plan Health Literacy in Germany origin, development and structure. J Public Health 31:905–915. 10.1007/s10389-021-01616-9

[17] Schaeffer D, Gille S, Hurrelmann K (2020) Implementation of the National Action Plan Health Literacy in Germany—lessons learned. IJERPH 17(12):4403. 10.3390/ijerph17124403 32575425 10.3390/ijerph17124403 PMC7345560

[18] Zok K (2014) Unterschiede bei der Gesundheitskompetenz. Ergebnisse einer bundesweiten Repräsentativ-Umfrage unter gesetzlich Versicherten. WIdO Monit 11(2):1–12

[19] Kirsch IS, Jungeblut A, Jenkins L, Kolstad A (2002) Adult literacy in America. A first look at the findings of the National Adult Literacy Survey. U.S. Department of. Education (Washington)

[20] U.S. Department of Education (2019) Adult literacy in the United States. https://nces.ed.gov/pubs2019/2019179/index.asp Zugegriffen: 14. Juni 2024

[21] Buddeberg K, Dutz G, Grotlüschen A, Heilmann L, Stammer C (2020) Low literacy in. RELA, Bd. 11. Results from the second German literacy survey, Germany, S 127–143 10.3384/rela.2000-7426.rela9147

[22] DeWalt DA, Berkman ND, Sheridan S, Lohr KN, Pignone MP (2004) Literacy and health outcomes. J Gen Intern Med 19:1228–1239. 10.1111/j.1525-1497.2004.40153.x 15610334 10.1111/j.1525-1497.2004.40153.x PMC1492599

[23] Rudd ER, Moeykens B, Colton TC (1999) Health and literacy. A review of medical and public health literature. Annual Review of Adult Learning and Literacy, New York

[24] Parker R, Ratzan SC (2010) Health literacy. A second decade of distinction for Americans. J Health Commun 2:20–33. 10.1080/10810730.2010.501094 10.1080/10810730.2010.501094 20845190

[25] Brach C, Keller D, Hernandez LM et al (2012) Ten Attributes of Health Literate Health Care Organizations. National Academy of. Sciences

[26] Bremer D, Klockmann I, Jaß L, Härter M, Knesebeck O, Lüdecke D (2021) Which criteria characterize a health literate health care organization?—a scoping review on organizational health literacy. BMC Health Serv Res 21:664. 10.1186/s12913-021-06604-z 34229685 10.1186/s12913-021-06604-z PMC8259028

[27] Nutbeam D (2000) Health literacy as a public health goal. A challenge for contemporary health education and communication strategies into the 21st century. Health Promot Int 15(3):259–267

[28] World Health Organization (2013) Regional Office for Europe. Health literacy. The solid facts. World Health Organization, Geneva

[29] HLS-EU Consortium (2012) Comparative report of health literacy in eight. EU, member states; The European Health Literacy Survey HLS-EU. Second Revised and Extended Version

[30] Stock S, Isselhard A, Jünger S et al (2022) DNVF Memorandum Gesundheitskompetenz (Teil II) – Operationalisierung und Messung von Gesundheitskompetenz aus Sicht der Versorgungsforschung. Gesundheitswesen 84:e26–e41. 10.1055/a-1807-0853 35472769 10.1055/a-1807-0853 PMC9050455

[31] Berkman ND, Sheridan SL, Donahue KE, Halpern DJ, Crotty K (2011) Low health literacy and health outcomes. an updated systematic review. Ann Intern Med 155:97–107. 10.1059/0003-4819-155-2-201107190-00005 21768583 10.7326/0003-4819-155-2-201107190-00005

[32] Rasu RS, Bawa WA, Suminski R, Snella K, Warady B (2015) Health literacy impact on national healthcare utilization and expenditure. Int J Health Policy Manag 4:747–755. 10.15171/ijhpm.2015.151 26673335 10.15171/ijhpm.2015.151 PMC4629700

[33] Tajdar D, Lühmann D, Fertmann R et al (2021) Low health literacy is associated with higher risk of type 2 diabetes: a cross-sectional study in Germany. Bmc Public Health 21:510. 10.1186/s12889-021-10508-2 33726714 10.1186/s12889-021-10508-2 PMC7962353

[34] Achstetter K, Köppen J, Haltaufderheide M, Hengel P, Blümel M, Busse R (2022) Health Literacy of People with Substitutive Private Health Insurance in Germany and Their Assessment of the Health System Performance According to Health Literacy Levels: Results from a Survey. Int J Environ Res Public Health. 10.3390/ijerph192416711 36554592 10.3390/ijerph192416711 PMC9778886

[35] Berens E‑M, Klinger J, Mensing M, Carol S, Schaeffer D (2022) Gesundheitskompetenz von Menschen mit Migrationshintergrund in Deutschland – Ergebnisse des HLS-MIG. Interdisziplinäres Zentrum für Gesundheitskompetenzforschung (IZGK). Universität Bielefeld, Bielefeld

[36] Rathmann K, Dadaczynski K, Okan O, Messer M (Hrsg) (2023) Gesundheitskompetenz. Springer, Pflege – Therapie – Gesundheit. Springer Berlin Heidelberg, Berlin, Heidelberg

[37] Schaeffer D, Berens E‑M, Vogt D et al (2021) Health literacy in Germany - findings of a representative follow-up survey. Dtsch Ärztebl Int 118:723–729. 10.3238/arztebl.m2021.0310 34551856 10.3238/arztebl.m2021.0310 PMC8820084

[38] Schaeffer D, Hurrelmann K, Bauer U, Kolpatzik K (2018) Nationaler Aktionsplan Gesundheitskompetenz. Die Gesundheitskompetenz in Deutschland stärken. KomPart, Berlin

[39] M-POHL (2023) Health literacy policies – how can they be developed and implemented? A guide for policy and decision makers. Vienna, International Coordination Center of M‑POHL at the Austrian National Public Health Institute

[40] Campos PA, Reich MR (2019) Political analysis for health policy implementation. Health Syst Reform 5:224–235. 10.1080/23288604.2019.1625251 31390295 10.1080/23288604.2019.1625251

[41] Ansell C, Sørensen E, Torfing J (2017) Improving policy implementation through collaborative policymaking. policy polit 45:467–486

[42] Goyal N, Howlett M (2019) Framework or metaphor? Analysing the status of policy learning in the policy sciences. J Asian Public Policy 12:257–273. 10.1080/17516234.2018.1493768

[43] Nilsen P (2015) Making sense of implementation theories, models and frameworks. Implement Sci 10:53. 10.1186/s13012-015-0242-0 25895742 10.1186/s13012-015-0242-0 PMC4406164

[44] Hower K, Pförtner T‑K, Pfaff H, Wensing M, Ansmann L (2021) Innovationen im Gesundheitswesen. In: Blättel-Mink B, Schulz-Schaeffer I, Windeler A (Hrsg) Handbuch Innovationsforschung. Springer, Wiesbaden, S 629–648

[45] Hurrelmann K, Klinger J, Schaeffer D (2022) Gesundheitskompetenz der Bevölkerung in Deutschland im Zeitvergleich der Jahre 2014 und 2020. Gesundheitswesen 85:314–322. 10.1055/a-1709-1011 35098501 10.1055/a-1709-1011 PMC11248117

[46] Okan O, Bollweg TM, Berens E‑M, Hurrelmann K, Bauer U, Schaeffer D (2020) Coronavirus-related health literacy: A cross-sectional study in adults during the COVID-19 infodemic in Germany. Int J Environ Res Public Health. 10.3390/ijerph17155503 32751484 10.3390/ijerph17155503 PMC7432052

[47] OECD (2023) Empowering through health literacy: Skills to navigate health information and make informed decisions. In: OECD – Organisation for economic cooperation and development (Hrsg) OECD Skills Outlook: Skills for a resilient green and digital transition. Chapter, Bd. 6. OECD Publishing, Paris, S 170–209

[48] Gugglberger L (2019) The multifaceted relationship between health promotion and health literacy. Health Promot Int 34:887–891. 10.1093/heapro/daz093 31755534 10.1093/heapro/daz093

[49] Wirtz MA, Soellner R (2022) Gesundheitskompetenz. Diagnostica 68:163–171. 10.1026/0012-1924/a000299

[50] HLS-PROF Konsortium. In: Professionelle Gesundheitskompetenz bei ausgewählten Gesundheitsprofessionen/-berufen. Ergebnisse der Pilotstudie HLS-PROF in der Schweiz. Hrsg), Bd. 2023. und Österreich. Careum, Hertie School, Universität Bielefeld und Gesundheit Österreich, Zürich, Berlin, Bielefeld, Wien, Deutschland

[51] World Health Organization (2023) Achieving well-being: a global framework for integrating well-being into public health utilizing a health promotion approach. World Health Organization, Geneva

